# Does Intra-Uterine Exposure to the Zika Virus Increase Risks of Cognitive Delay at Preschool Ages? Findings from a Zika-Exposed Cohort from Grenada, West Indies

**DOI:** 10.3390/v15061290

**Published:** 2023-05-30

**Authors:** Michelle Fernandes, Roberta Evans, Mira Cheng, Barbara Landon, Trevor Noël, Calum Macpherson, Nikita Cudjoe, Kemi S. Burgen, Randall Waechter, A. Desiree LaBeaud, Karen Blackmon

**Affiliations:** 1 MRC Lifecourse Epidemiology Centre, Faculty of Medicine, University of Southampton, Southampton SO16 6YD, UK; 2 Human Development and Health Academic Unit, Faculty of Medicine, University of Southampton, Southampton SO16 6YD, UK; 3 Nuffield Department of Women’s Productive Health, John Radcliffe Hospital, University of Oxford, Oxford OX3 9DU, UK; 4 Caribbean Center for Child Neurodevelopment, Windward Islands Research and Education Foundation, St. George P.O. Box 7, Grenada; 5 Department of Pediatrics, Infectious Disease Division, Stanford University School of Medicine, Stanford, CA 94304, USA; 6 Department of Physiology, Neuroscience, and Behavioral Science, School of Medicine, St. George’s University, St. George P.O. Box 7, Grenada; 7 Department of Psychiatry and Psychology, Mayo Clinic, Jacksonville, FL 32224, USA

**Keywords:** Zika virus, intra-uterine Zika virus exposure, normocephaly, neurodevelopment, cognition, executive function, vision, neurodevelopmental profiles

## Abstract

Maternal infection with Zika virus (ZIKV) is associated with a distinct pattern of birth defects, known as congenital Zika syndrome (CZS). In ZIKV-exposed children without CZS, it is often unclear whether they were protected from in utero infection and neurotropism. Early neurodevelopmental assessment is essential for detecting neurodevelopmental delays (NDDs) and prioritizing at-risk children for early intervention. We compared neurodevelopmental outcomes between ZIKV-exposed and unexposed children at 1, 3 and 4 years to assess exposure-associated NDD risk. A total of 384 mother–child dyads were enrolled during a period of active ZIKV transmission (2016–2017) in Grenada, West Indies. Exposure status was based on laboratory assessment of prenatal and postnatal maternal serum. Neurodevelopment was assessed using the Oxford Neurodevelopment Assessment, the NEPSY^®^ Second Edition and Cardiff Vision Tests, at 12 (*n* = 66), 36 (*n* = 58) and 48 (*n* = 59) months, respectively. There were no differences in NDD rates or vision scores between ZIKV-exposed and unexposed children. Rates of microcephaly at birth (0.88% vs. 0.83%, *p* = 0.81), and childhood stunting and wasting did not differ between groups. Our results show that Grenadian ZIKV-exposed children, the majority of whom were without microcephaly, had similar neurodevelopmental outcomes to unexposed controls up to at least an age of 4 years.

## 1. Introduction

The timely achievement of cognitive milestones in the first few years of life is the most important predictor of cognitive outcomes during school age and adolescence [1]. A recent neuroimaging study demonstrated that most local and global brain growth metrics peak before 3 years of age, with some regions peaking at 5–6 years and other regions peaking in late childhood (+9 years) [2]. Early assessment of cognitive functions in children with intra-uterine Zika virus (ZIKV) exposure can identify individuals at risk for delay and prioritize them for early interventions. However, not all children with prenatal ZIKV exposure are at risk. Although ZIKV targets neural stem cells in the developing brain, neurological manifestations are not apparent at birth in the majority of ZIKV-exposed children [3,4].

Children born to women infected with ZIKV during pregnancy have a 5 to 14% risk of congenital Zika syndrome (CZS) [5] and 4 to 6% risk of microcephaly [6]. Among the birth defects associated with CZS, the presence and severity of microcephaly predicts neurodevelopmental delays (NDDs) during early childhood [7] along a “gradient of risk of developmental delay according to head circumference” [4]. Nevertheless, 94 to 96% of ZIKV-exposed children do not present with microcephaly at birth [3,4,8]. Some reports caution against the absence of microcephaly in ZIKV-exposed children being a proxy marker for presumed normal development [9,10,11]. One study reported abnormal neurology (i.e., abnormalities of tone, ataxia, dyskinesia and irritability) in 18 of 19 normocephalic ZIKV-exposed children during the first year of life [12]. Nevertheless, data from a large longitudinal study, led by Brazil’s Microcephalic Epidemic Research Group, showed that ZIKV-exposed children without microcephaly were not at increased risk of neurodevelopmental delay on standardized neurodevelopmental testing at 27–30 months when compared with their unexposed peers [4]. We have previously reported similar findings from a large Grenadian cohort at 24 months of age [3]. However, few data on the medium- to long-term risks of subtle brain and ocular injury exist in these children: were they protected from the effects of ZIKV neurotropism or do cognitive delays manifest as they age? [3] An understanding of their neurodevelopmental trajectories is of substantial public health significance and has potentially important implications for the physical health, mental health and social outcomes of these children throughout the course of their lives.

Our objective was to extend the understanding of neurodevelopment in normocephalic ZIKV-exposed children by longitudinally tracking neurodevelopmental outcomes and anthropometrics from birth to 48 months of age in a cohort of ZIKV-exposed children and a parallel group of unexposed controls born during a period of active ZIKV transmission in Grenada, West Indies [13]. We hypothesized that the absence of cognitive delays and microcephaly at age 1 year is a positive harbinger of normal neurocognitive development in ZIKV-exposed children. In the present study, we (1) compared neurodevelopmental outcomes and rates of microcephaly between ZIKV-exposed children and unexposed controls at 1, 3 and 4 years; and (2) examined whether environmental and perinatal factors that increase and decrease NDD risk differ between the ZIKV-exposed and unexposed groups. By carrying these analyses out, we aimed to examine whether the neurodevelopmental profiles from birth to 48 months differ between ZIKV-exposed and unexposed children, and sought to clarify whether the differences, if any, in these profiles are attributed to ZIKV exposure, socio-environmental influences, or both.

## 2. Materials and Methods

### 2.1. Study Population and Procedures

This ambispective, population-based cohort study followed 388 children born to 384 mothers during a period of active ZIKV transmission in Grenada, West Indies. The children were followed from birth to 4 years of age.

Mother–child dyads were enrolled between April 2016 and March 2017 at public health centres throughout Grenada [3].

**Prenatal cohort:** One hundred and fifty three women were enrolled during pregnancy. Maternal serum was collected at enrolment (median weeks of pregnancy when serum was collected: 28.0 weeks; min: 3.6; max: 41.9).

**Postnatal cohort:** Two hundred and thirty one mothers were enrolled post-birth, at which time maternal serum was collected (median time since birth: 5.4 months; min: 0.2; max: 23.1).

Data on perinatal outcomes and anthropometric measurements were collected soon after birth. Neurodevelopmental outcomes and anthropometric measurements were collected at 12, 36 and 48 months post-birth. Information on child health and socio-environmental indicators were collected at each time point. Data were collected from medical records as well as via direct interviews with the primary caregiver.

### 2.2. Laboratory Testing

Maternal serum samples were initially assessed for flavivirus exposure using indirect IgG capture enzyme-linked immunosorbent assay (ELISA) with pooled dengue virus (DENV) antigen [14]. The sample then underwent a multiplexed assay on a nanostructure plasmonic gold (pGOLD) platform (Nirmidas Biotech, Palo Alto, CA, USA) at Stanford University to cross-validate the ELISA results and to distinguish ZIKV from DENV exposure [15]. The pGOLD IgG immunoassay has a demonstrated 90% sensitivity and 98% specificity for ZIKV during the convalescent phase [15]. Many mother–infant pairs were enrolled in the study after delivery; therefore, serology testing was necessary to determine prior ZIKV exposure. We classified mothers as “infected” during pregnancy only if serum was collected during the prenatal period. Mothers who had positive serology for ZIKV during the postnatal period only were classified as “possibly exposed but unconfirmed” and were not included in further analyses. However, if they tested negative, we did include them in the study as “uninfected.” pGold testing was used to retrospectively determine whether mothers and infants had been exposed to ZIKV. pGold is a sensitive ELISA test that confirms both IgM and IgG for both Zika and dengue viruses [3]. This test is able to distinguish between these two closely related flaviviruses and is specifically designed to minimise cross-reactivity [15].

Child ZIKV exposure status (exposed vs. unexposed) was classified according to the results of the maternal serum pGOLD IgG immunoassay (infected vs. uninfected). Children were classified as ZIKV-exposed if they were born to mothers who had positive pGOLD results for ZIKV, with avidity testing showing infection in the past 6 months [3]. Children were classified as ZIKV-unexposed if they were born to mothers who had negative pGOLD results for ZIKV [3] during the prenatal and postnatal period.

### 2.3. Inclusion Criteria

For the 1, 3 and 4 years assessments, we used the previously published definitions of ZIKV-exposed children and unexposed controls [3]. Briefly, children were included in the ZIKV-exposed group if they were born to mothers classified as ‘ZIKV-Infected’ during pregnancy based on positive prenatal laboratory results for ZIKV, with avidity testing showing infection in the past 6 months. Children were included in the unexposed group if they were born to mothers who were classified as ‘ZIKV-Uninfected’ during pregnancy, based on prenatal and postnatal negative laboratory results for ZIKV. Children were excluded from both groups if they completed <50% of the items on the OX-NDA and NEPSY-II. A total of 32 children were excluded at years 1 (*n* = 26), 3 (*n* = 5) and 4 (*n* = 1) due to <50% completion rate on the measures of neurodevelopment.

### 2.4. Neurodevelopmental and Vision Assessments

Neurodevelopmental and vision assessments were administered by local research staff with undergraduate or graduate degrees in Psychology or Public Health who were trained and standardized in the assessments. All assessors were fluent in the local dialect and masked to child exposure status.

#### 2.4.1. The Oxford Neurodevelopmental Assessment (OX-NDA)

The OX-NDA is a comprehensive, rapid assessment of cognition, motor skills, language and (positive and negative) behaviour for children aged 10–14 months [16,17]. Its 37 items are administered in approximately 25 min using a combination of psychometric techniques (direct administration, concurrent observation and caregiver reports). A mixed methodology approach consisting of direct neurodevelopmental testing, and caregiver- and observer-rated reports was specifically selected so as to minimise the risks of reporting and recall bias commonly encountered in caregiver interviews while acknowledging that children might perform differently in artificial testing environments compared to in familiar settings. Children’s performance on the OX-NDA is scored across a spectrum of abilities, rather than on a predefined checklist and, therefore, affords a wider description of a child’s faculties. It has demonstrated satisfactory agreement with the BSID, third edition (BSID-III) (interclass correlation coefficients 0.63 and 0.68, *p* < 0.001 for cognitive and motor outcomes, and 0.30, *p* < 0.04 for language outcomes, with little to no bias on Bland–Altman analysis); satisfactory internal consistency (Cronbach’s alpha 0.56–0.81); and high levels of inter-rater (k = 0.80–0.96, 95% CI 0.78–0.97) and test–retest reliability (k = 0.85–0.94, 95% CI 0.80–0.95) across all domains [16]. For each child, the OX-NDA’s raw domain scores were converted to standardized scores (range = 0–100, mean = 50) and compared to its thresholds for moderate-to-severe delay (≤60 for cognitive and language delays, ≤74 for motor delay) [16].

The OX-NDA is designed for use across socioeconomic groups and populations. Its kit consists of common household items encountered across the world. In Grenada, the OX-NDA was customized for cultural relevance and acceptability using the WHO Mental Health Initiative translation guidelines [18].

#### 2.4.2. The Developmental Neuropsychological Assessment, Second Edition (NEPSY-II)

The NEPSY-II is a standardized, psychometrically valid neuropsychological assessment for preschoolers, children, and adolescents [19]. It consists of 32 subtests measuring executive function, language, memory and learning, sensorimotor functioning, visuospatial processing, and social perception in children aged 3 to 16 years [20]. In our cohort, we used 10 of the 32 available NEPSY-II subtests to assess children at 3 (36–47 months) and 4 (48–54 months) years of age. The selected subtests were mapped onto the domains of cognition, motor, language, and personal social development. NEPSY-II scores for each domain were categorized as follows: “Well Below Expected” (scaled score of 1–3), “Below Expected” (scaled score of 4–5), “Slightly Below Expected” (scaled score of 6–7), “As Expected” (scaled score of 8–12), and “Well Above Expected” (scaled score of 13–19) [20]. Children scoring in the “Well Below Expected”, “Below Expected”, or “Slightly Below Expected” categories were classified as having delayed neurodevelopment in that domain. Children scoring “As Expected” or “Well Above Expected” were classified as having expected neurodevelopment in that domain. Some degree of error is expected in classifying children as delayed given that the NEPSY-II normative reference sample was collected outside of the Caribbean (in the U.S.), which can lead to overestimation of developmental delay. However, this error equally applies to both the ZIKV-exposed and unexposed groups and, therefore, does not affect group comparisons.

#### 2.4.3. The Cardiff Tests of Vision

Visual acuity and contrast sensitivity were assessed in the cohort’s children at 1, 3 and 4 years using the Cardiff Tests [21,22,23]. The Cardiff Tests are valid and reliable measures of binocular vision in young children. Their administration and results are not influenced by coexisting disturbances in language or cognition, and are independent of cultural biases. Their norms have been applied for clinical purposes [21,22,23]. Their administration takes 5–7 min. Visual acuity (in LogMar) and contrast sensitivity (in %) are measured in quick succession and, measured together, are a more robust assessment of the integrity and functioning of the entire visual pathway than either test alone. In our cohort, we applied the administration protocol for the Cardiff Tests of visual acuity and contrast sensitivity as published by the INTERGROWTH-21st Project (freely available at available at https://www.intergrowth21.org.uk (accessed on 1 March 2023)). Vision in all children was assessed at a working distance of 50 cm.

### 2.5. Anthropometric Measures and Head Circumference Classification

Weight and length were measured at 1, 3 and 4 years of age using the WHO Multicentre Growth Reference Study protocols [24,25]. Measurements were undertaken by three independent, trained and standardized researchers and the mean of these measurements were used for group comparisons and for comparisons with the WHO’s child growth standards. Wasting was defined as more than two standard deviations below the median weight for sex and age and stunting was defined as more than two standard deviations below the median length or height for sex and age on the WHO child growth standards [26].

Serial measurements of occipitofrontal HC at birth and 1 year of age were performed to ensure that none of the children developed late onset microcephaly. Normocephaly was defined as occipitofrontal HC between the 4th and 96th percentile, borderline microcephaly was defined as between 1st and 3rd percentile, and microcephaly was defined as less than 1st percentile, for sex and age in accordance with WHO child growth standards [26].

### 2.6. Health and Socio-Environmental Outcomes

Information on perinatal and birth outcomes was obtained from hospital records and through a structured clinical interview at the 1-year follow-up visit. Information on child health outcomes, household and socio-environmental characteristics was collected at each follow-up visit (birth, 1, 3 and 4 years) using structured caregiver interviews. These characteristics included measures of food security (USDA Food Security Questionnaire) [27], maternal mental health (General Health Questionnaire, GHQ-12) [28], maternal social support (Social Support Questionnaire) [29], and household environment (Home Environment Questionnaire) [30].

### 2.7. Statistical Analysis

Maternal and child data across the follow-up visits were entered into a secure, online database Research Electronic Data Capture (REDCap) [31]. Statistical analyses were performed using the Statistical Package for the Social Sciences (SPSS), V.28. All hypothesis’ testing was two-sided with a significance threshold of *p* < 0.05.

Participant characteristics, including socio-demographic measures, perinatal factors, and child anthropometrics, were compared between the ZIKV-exposed and unexposed groups using chi-square tests or Fisher’s exact tests for categorical variables, and independent samples *t*-tests for continuous variables. Attrition analyses of socio-demographic measures were performed across timepoints between mother–child dyads who completed outcome assessments and those who were lost to follow-up.

Rates of neurodevelopmental delay for the OX-NDA cognition, motor and language domains and the NEPSY-II cognition, motor, language, and personal social domains were compared between the ZIKV-exposed and unexposed groups using chi-square tests. Continuous OX-NDA and NEPSY-II scores across all domains were compared between groups using independent samples *t*-tests. Cardiff visual acuity and contrast sensitivity scores were compared between groups using independent samples *t*-tests, and rates of low visual acuity and low contrast sensitivity were compared between groups using Fisher’s exact tests.

### 2.8. Ethical Approval

The study was approved by the Institutional Review Boards of St. George’s University, Grenada, West Indies (IRB#16061) and Stanford University, USA (IRB#s 37004 and 45242), and granted research clearance by the Grenada Ministry of Health. Mothers provided written informed consent for themselves and on behalf of their participating children.

## 3. Results

**Figure 1:**
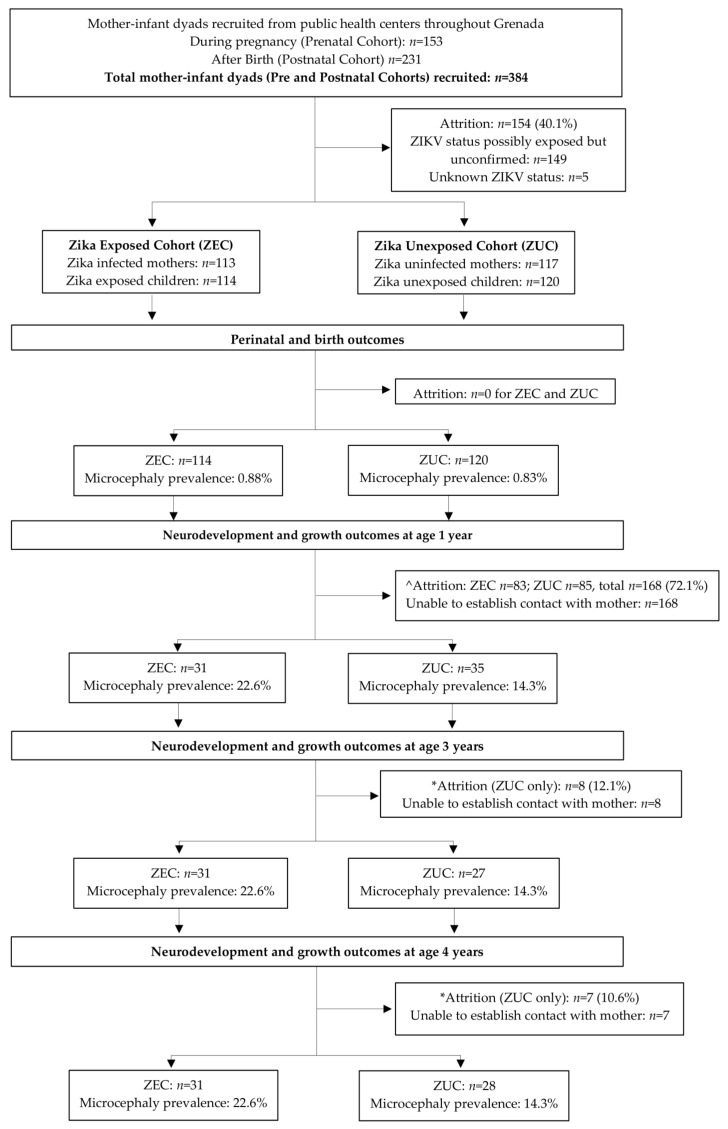
Flowchart presenting the patients enrolled in the study. ^ Attrition rates estimated as difference between birth and 1 year sample sizes. * Attrition rates estimated as difference between 3 and 1 year, and 4 and 1 year sample sizes, respectively.

The flowchart with patients enrolled in the study is presented in Figure 1. Of the 388 children (384 mothers) recruited, 154 were excluded from analyses because they tested positive for ZIKV on postnatal serology alone (*n* = 149), or their exposure status was indeterminable (*n* = 5). Of the eligible mother–infant dyads, 113 were classified as ZIKV-infected during pregnancy (mother) and 114 as ZIKV-exposed (child), and 117 were classified as ZIKV uninfected (mother) and 120 as unexposed (child). Of these, 31 ZIKV-exposed children completed neurodevelopmental assessments at 1, 3 and 4 years, and 35, 27 and 28 unexposed children completed these assessments at 1, 3 and 4 years of age, respectively. Despite high rates of attrition (72% for ZIKV-exposed children, and 70.8–77.5% for unexposed children; attrition rates presented are calculated against the 1-year assessment), there were no differences in socio-environmental indicators between the children who completed the neurodevelopmental assessments and those who were lost to follow-up.

### 3.1. Participant Characteristics

The socio-demographic, birth and growth characteristics of the cohort, collected at 1 year of age, are presented in Table 1. Overall, 60% of the cohort was male (*n* = 40); the distribution of male and female children did not differ significantly between the ZIKV-exposed and unexposed groups. The overall risk of perinatal morbidity in the cohort was low: 13% of children were born preterm; 11% had reported complications during birth; and 4.5% required resuscitation at birth. Median APGAR scores at 1 min were 8.00 (2.00–7.00) and 8.00 (4.00–9.00) for ZIKV-exposed and unexposed children, respectively. Median gestational age at birth was identical for both groups (40.0 weeks, 26.00–42.00). Duration of exclusive breastfeeding and age at weaning did not differ significantly between the two groups. Most mothers and their partners had completed secondary school education. There were no differences in socio-demographic or perinatal factors between ZIKV-exposed and unexposed children (Table 1).

### 3.2. Comparisons between ZIKV-Exposed and Unexposed Children at 1, 3 and 4 Years 

#### 3.2.1. Neurodevelopmental Scores and Prevalence of Neurodevelopmental Delay

**Figure 2:**
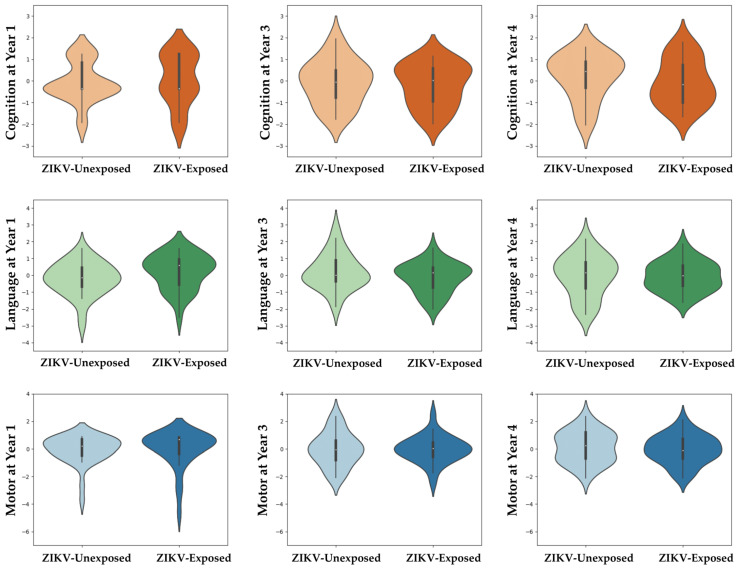
Neurodevelopment scores across domains at ages 1, 3 and 4 years between ZIKV-exposed and unexposed children. Violin plots show no group differences between ZIKV-exposed and unexposed children in any neurodevelopmental domain at any time point. Scores were centred around the mean for each age group (z = (x − μ)/σ) to adjust for scale differences between the OX-NDA (year 1) and the NEPSY (years 3 and 4).

OX-NDA scores at age 1 year, and NEPSY-II scores at ages 3 and 4 years, across domains did not differ between ZIKV-exposed and unexposed children (Figure 2, Table 2 and Table 3). Cognition at age 1 year significantly differed between the groups (*p* = 0.048). The unexposed group had a higher rate of cognitive delay; however, this difference did not persist at ages of 3 and 4 years.

The rates of any and no delay for cognitive, motor and language domains (and personal–social for the NEPSY-II) between ZIKV-exposed and unexposed children at ages 1, 3 and 4 years are presented in Table 4. There were no differences in the rates of delay between groups across domains at ages 1, 3 and 4 years.

#### 3.2.2. Visual Acuity and Contrast Sensitivity

The visual acuity and contrast sensitivity scores, and rates of low vision, did not differ between ZIKV-exposed and unexposed children, measured at ages of 3 and 4 years (Table 5).

#### 3.2.3. Microcephaly and Growth Outcomes

Weight, length and head circumference were similar between the two groups at birth and at 1 year of age (Table 1). Rates of childhood stunting and wasting did not differ between groups (Table 1).

At birth, the rates of microcephaly did not differ between ZIKV-exposed (0.88%) and unexposed (0.83%) children, and microcephaly was reported only in one child in each group. Rates of microcephaly at 1 year of age were higher than those recorded at birth in both groups (22.6% vs. 14.3%); these, however, did not differ significantly between ZIKV-exposed and unexposed children.

## 4. Discussion

In this study, weight, length and head circumference were similar between ZIKV-exposed and unexposed children at birth and at 1 year of age, and rates of childhood stunting, wasting, and microcephaly did not differ between groups. We found no evidence of neurodevelopmental delays in cognition, motor, language, behaviour, personal–social and vision outcomes in ZIKV-exposed children at 1, 3 and 4 years of age, relative to a parallel group of socio-demographically similar but unexposed children. Although there was a marginal difference in NDD rates for the cognition domain at 1 year of age, there were higher rates of delay in the unexposed, rather than the ZIKV-exposed group. This difference was no longer apparent at 3 and 4 years of age. Overall, the findings suggest that the absence of NDDs and microcephaly at age 1 year is a positive harbinger of normal neurocognitive development in ZIKV-exposed children. However, we do acknowledge that our study was observational in design and cannot therefore (dis)prove causation.

Our findings are consistent with those previously reported for 2-year-old neurodevelopmental outcomes in the same cohort: no differences in 2-year cognitive, motor, language or behaviour scores, or rates of delay were reported between ZIKV-exposed and unexposed children [3]. A population-based cohort study in three French territories of the Caribbean (French Guiana, Guadeloupe, and Martinique) yielded similar findings at 24 months—no discernible neurodevelopmental differences between ZIKV-exposed and ZIKV-unexposed toddlers [32]. The Microcephaly Epidemic Research Group found that prenatal ZIKV exposure was not associated with increased rates of developmental delay in normocephalic Brazilian children, aged 6 to 42 months [4]. Moreover, a cohort study from French Polynesia found that maternal ZIKV infection was not associated with an excess burden of developmental delay during early childhood [33]. Data from the U.S. Zika Pregnancy and Infant Registry show that among children with confirmed prenatal Zika-exposure but without Zika-associated birth defects, the rate of confirmed or possible NDD was 7.4% up to 36 months of age, which is considered to be within the expected general population rate [unpublished personal data] [34]. Nevertheless, developmental delays, particularly in cognitive and language domains, have been previously reported in normocephalic ZIKV-exposed children [34,35,36,37] and authors have cautioned against normocephaly being used as a proxy marker for reassurance of typical neurodevelopment [12]. In one Brazilian study, lower head circumference for age was found to be a predictor of neurodevelopmental delay in normocephalic ZIKV-exposed children at 6 months [38]. However, many of the studies reporting associations between maternal ZIKV infection and delays in development during early childhood are limited by the absence of local unexposed controls and small sample sizes without longitudinal follow-up [3]. These factors may result in an over-estimation of risk, especially when children from ZIKV-exposed cohorts are compared with those from socio-demographically and culturally disparate normative reference groups. By including a local unexposed control group and demonstrating that this group had similarly low rates of perinatal morbidity and socio-environmental adversity to the ZIKV-exposed group, the current study overcame this limitation. By following these children up to 48 months of age, our approach revealed that the ZIKV-exposed children in our cohort are performing on par with their unexposed peers across measures of cognitive, motor, language, vision, behavioural and personal–social development at 1, 3 and 4 years of age. However, further surveillance of these children into school age and adolescence is needed to determine whether they continue to show neurodevelopment similar to that of their unexposed peers [39,40].

One major limitation of our study was its reliance on serologic (IgM/IgG) testing for the identification of maternal ZIKV infection during pregnancy. Although nucleic acid amplification tests (NAATs) provide precise evidence on the actual presence of infection during the testing period, many mother–infant pairs were enrolled in the study after delivery; therefore, serology testing was necessary to determine prior ZIKV exposure. We classified mothers as infected during pregnancy (*n* = 113) only if serology was collected during the prenatal period. Mothers who had serum collected during the postnatal period only were classified as “possibly exposed but unconfirmed” (*n* = 149) and were not included in further analyses. However, if they tested negative, we did include them in the study as “uninfected”. Second, as our sample size was small due to high rates of attrition (approximately 50% between 1 and 4 year assessments), it is important to consider the likelihood of type II error when interpreting our findings. Nevertheless, our final sample size of 234 and 59, respectively, at the 1- and 4-year follow-ups is larger than and comparable with the 2-year follow-up sizes of approximately *n* = 60 of many ZIKV-exposed birth cohorts [40]. Third, we were unable to confirm maternal ZIKV infection with laboratory testing during pregnancy in 149 women, resulting in the exclusion of these mother–child dyads from follow-up at subsequent ages. Fourth, we did not include neuroimaging as an outcome measure; therefore, we were unable to ascertain whether ZIKV-associated imaging findings commonly reported among normocephalic ZIKV-exposed children were also present in our cohort [10,36]. However, previous studies report that the agreement between the structural abnormalities on neuroimaging and functional and/or neurodevelopmental abnormalities on psychometric testing in ZIKV-exposed children is not consistent [36]. Fifth, by including all pregnant mothers, regardless of symptom status, we were unable to map the timing of congenital ZIKV exposure onto the embryological timeline of brain development. We are therefore unable to determine whether the low rates of neurodevelopmental delays observed in our cohort are due to infection occurring later in pregnancy when the protective effects of mature villous trophoblasts are established [41,42], or other yet to be identified in and ex utero protective factors [43]. Finally, it is important to note that maternal infection does not imply ZIKV infection in the developing infant. It is possible that ZIKV-exposed infants in our sample may have been protected from ZIKV infection. Foetal infection would need to be demonstrated via amniocentesis, which is not clinically recommended due to associated risk of pregnancy complications, the transient presence of ZIKV RNA in developing fetuses, which increases risk of false negatives, and lack of evidence that it predicts the risk for congenital Zika syndrome abnormalities [44].

In this study, ZIKV-exposed and unexposed children were similar in socio-demographic, socio-environmental, perinatal and growth characteristics between 0 and 4 years of age, which increases our confidence in findings of no neurodevelopmental difference between the two groups. Additionally, we recruited participants from public health centres in every parish to increase the likelihood that our sample was socio-demographically representative of the larger Grenadian population. Randomized controlled designs are necessary to causally examine the consequences of viral exposure on neurodevelopment but this is often not feasible during pandemics. Prospective and ambispective birth cohort studies offer the next best opportunity to address complex etiological questions about prenatal exposures and neonatal outcomes, even though they are unable to prove (or disprove) causal links. A further strength of our study was the follow-up of ZIKV-exposed children into preschool age, which allowed us to examine whether NDDs manifest as the demand for more complex cognitive information processing increases as children age. There are few cohorts that have followed ZIKV-exposed children beyond 2 years of age [4,33,36]. Nevertheless, we acknowledge that recent neuroimaging studies have shown that the growth and development of certain brain regions peak after the preschool period [2], in middle and late childhood; therefore, the long-term follow-up of such cohorts is needed to ascertain whether neurodevelopment in ZIKV-exposed children remains typical at these ages (and beyond) or atypical neurodevelopmental and neurological outcomes manifest as children age. Finally, as ZIKV transmission is mosquito-borne, endemic areas are often at high risk of other mosquito-borne viral infections which may/may not independently impact early brain development. In our study, we used the pGOLD platform [15] (specifically designed to minimise cross-reactivity within the *Flaviviridae*) to distinguish ZIKV from DENV exposure, which is common in Grenada. Our study adds to the growing body of literature that ZIKV-exposed children without neurologic manifestations at birth or during infancy do not appear to be at elevated risk for developmental delay during the preschool years [4,33].

## 5. Conclusions

Longitudinal tracking of ZIKV-exposed children alongside a parallel group of socio-demographically similar, unexposed children revealed no major delays in cognition, motor skills, language, vision, behaviour or personal–social skills at 1, 3 and 4 years of age, with no differences in neurodevelopmental scores between the two groups. Additionally, early life factors associated with higher rates of NDDs did not vary systematically between the groups. Considering that we could not confirm fetal ZIKV infection in our sample, it is possible that many of the exposed children were protected in utero from vertical transmission. Nevertheless, further longitudinal follow-up of ZIKV-exposed children, from different populations and geographies, is needed to confirm that these children continue to develop along typical neurodevelopmental trajectories into school age and adolescence.

## Figures and Tables

**Table 1:** Participant characteristics and attrition analyses.

Participant Characteristics	Pooled Sample: Birth and 1 Year	ZIKV-Exposed (Birth and 1 Year)	Unexposed (Birth and 1 Year)	Test Statistic, *p*	Pooled Sample: 3 Years Follow-Up (N or Mean (SD)	Pooled Sample: 4 Years Follow-Up (N or Mean (SD)
	N (%) or Mean (SD)	N (%) or Mean (SD)	N (%) or Mean (SD)		Test Statistic ^g^, *p*	Test Statistic ^h^, *p*
**Sex** Male Female	66 (0.50)	16 (40.0) 15 (57.7)	24 (60.0) 11 (42.3)	X^2^ = 1.98 *p* = 0.21	29	28
					X^2^ = 0.80 *p* = 0.37	X^2^ = 0.48 *p* = 0.49
**Age at 1 year assessment**	15.00 (2.29)	15.52 (1.99)	14.54 (2.47)	*t* = 1.75 *p* = 0.08	41.24 (3.56)	50.36 (1.16)
					*t* = 1.15 *p* = 0.26	*t* = 0.08 *p* = 0.94
**Birth Outcomes**						
**Prematurity** Premature Full-term	52 (0.35)	3 (42.9) 21 (46.7)	4 (57.1) 24 (53.3)	^^ *p* = 1.00	24	27
					X^2^ = 0.06 *p* = 0.80	X^2^ = 0.63 *p* = 0.43
**Delivery type** Caesarean Vaginal	66 (0.42)	6 (40.0) 25 (49.0)	9 (60.0) 26 (51.0)	X^2^= 0.38 *p* = 0.57	29	28
					X^2^ = 0.56 *p* = 0.76	X^2^ = 1.06 *p* = 0.59
**Delivery complications** ^a^ Yes No	65 (0.31)	3 (42.9) 28 (48.3)	4 (57.1) 30 (51.7)	^^ X^2^= 0.07 *p* = 1.00	29	28
					X^2^ = 0.01 *p* = 0.94	X^2^ = 0.90 *p* = 0.34
**Neonatal resuscitation** ^b^ Yes No	66 (0.05)	2 (66.7) 29 (46.0)	1 (33.3) 34 (54.0)	^^ *p* = 0.59	29	28
					X^2^ = 0.07 *p* = 0.79	X^2^ = 0.12 *p* = 0.73
**Neonatal complications** ^c^ Yes No	63 (0.37)	5 (50.0) 25 (47.2)	5 (50.0) 28 (52.8)	X^2^= 0.03 *p* = 1.00	28	28
					X^2^ = 0.01 *p* = 0.92	X^2^ = 0.02 *p* = 0.90
**Congenital abnormalities** ^d^ Yes No	66 (0.12)	1 (100.0) 30 (46.2)	0 (0.0) 35 (53.8)	^	29	28
					X^2^ = 1.64 *p* = 0.20	X^2^ = 0.12 *p* = 0.73
**Chromosomal abnormalities** Yes No	48 (0.14)	0 (0.0) 23 (48.9)	1 (100.0) 24 (51.1)	^	11	5
					^	^
**APGAR at 1 min**	7.92 (1.42)	7.74 (1.86)	8.09 (0.85)	*t* = −0.94 *p* = 0.35	8.28 (0.92)	7.89 (1.19)
					*t* = −1.05 *p* = 0.30	*t* = 0.57 *p* = 0.57
**Maternal age at delivery**	29.30 (6.74)	28.68 (6.98)	29.88 (6.56)	*t* = 0.71 *p* = 0.48	27.85 (5.93)	28.56 (6.69)
					*t* = 0.98 *p* = 0.33	*t* = 0.67 *p* = 0.50
**Gestational age at birth**	39.05 (2.17)	39.17 (1.90)	38.43 (2.95)	*t* =-1.05 *p* = 0.30	38.71 (2.71)	39.48 (1.52)
					*t* = 0.46 *p* = 0.65	*t* = −1.10 *p* = 0.28
**Total duration of exclusive breastfeeding period length (months)**	15.80 (7.53)	14.67 (7.37)	17.50 (8.54)	*t* = −0.56 *p* = 0.59	21.00 (8.19)	15.60 (7.25)
					*t* = −1.67 *p* = 0.12	*t* = −0.50 *p* = 0.62
**Age at weaning**	12.33 (7.06)	13.30 (6.83)	11.56 (7.30)	*t* = 0.91 *p* = 0.37	13.11 (8.60)	13.04 (6.94)
					*t* = −0.00 *p* = 0.99	*t* = −0.02 *p* = 0.98
**Growth Outcomes at Birth**						
**Weight (kg)**	3.07 (0.55)	3.11 (0.49)	3.04 (0.61)	*t* = 0.41 *p* = 0.69	3.19 (0.56)	3.26 (0.66)
					*t* = 0.08 *p* = 0.93	*t* = −0.55 *p* = 0.58
**Weight WHO classification** Wasted Normal	33 (0.24)	0 (0.0) 14 (45.2)	2 (100.0) 17 (54.8)	^^ *p* = 0.49	18	21
					X^2^ = 4.99 *p* = 0.03	X^2^ = 1.41 *p* = 0.24
**Length (cm)**	47.96 (3.89)	47.97 (2.63)	47.95 (4.73)	*t* = 0.01 *p* = 0.99	46.03 (7.00)	49.27 (2.95)
					*t* = 1.13 *p* = 0.27	*t* = −1.60 *p* = 0.12
**Length WHO classification** Stunted Normal	34 (0.41)	4 (57.1) 11 (40.7)	3 (42.9) 16 (59.3)	^^ *p* = 0.67	18	22
					X^2^ = 0.17 *p* = 0.68	X^2^ = 2.63 *p* = 0.11
**Head circumference (cm)**	33.18 (1.75)	33.29 (1.65)	33.09 (1.85)	*t* = 0.37 *p* = 0.71	33.04 (1.92)	33.61 (1.63)
					*t* = 0.95 *p* = 0.35	*t* = −0.55 *p* = 0.59
**Head circumference classification at birth** Normocephalic Borderline microcephalic ^e^ Microcephalic ^f^ Macrocephalic	48 (0.47)	19 (45.2) 1 (25.0) 1 (50.0) 0 (0.0)	23 (54.8) 3 (75.0) 1 (50.0) 0 (0.0)	^^ X^2^= 0.64 *p* = 0.81	23	22
					X^2^ = 2.28 *p* = 0.32	X^2^ = 2.35 *p* = 0.50
**Growth Outcomes at Age 1 Year**						
**Weight (kg)**	6.70 (2.56)	6.63 (1.97)	6.76 (3.00)	*t* = −0.21 *p* = 0.83	7.25 (2.47)	7.13 (1.84)
					*t* = 0.87 *p* = 0.39	*t* = 1.19 *p* = 0.24
**Weight WHO classification** Wasted Normal	65 (0.44)	3 (60.0) 27 (45.8)	2 (40.0) 32 (54.2)	^^ *p* = 0.66	9	7
					X^2^ = 0.67 *p* = 0.41	X^2^ = 0.40 *p* = 0.53
**Length (cm)**	63.93 (8.42)	63.90 (7.99)	62.71 (8.70)	*t* =-0.58 *p* =0.57	63.87 (9.14)	64.11 (6.39)
					*t* = 1.207 *p* = 0.23	*t* = 1.36 *p* = 0.18
**Length WHO classification** Stunted Normal	66 (0.29)	2 (33.3) 29 (48.3)	4 (51.7) 31 (66.7)	^^ *p* = 0.67	11	7
					X^2^ = 0.70 *p* = 0.40	X^2^ = 1.51 *p* = 0.22
**Head circumference (cm)**	40.21 (3.83)	40.59 (4.41)	39.87 (3.27)	*t* = 0.75 *p* = 0.46	41.38 (4.40)	41.13 (2.94)
					*t* = 0.65 *p* = 0.52	*t* = 1.07 *p* = 0.29
**Head circumference classification at 1** Normocephalic Borderline microcephalic ^e^ Microcephalic ^f^ Macrocephalic	66 (0.89)	23 (47.9) 0 (0.0) 7 (58.3) 1 (100.0)	25 (52.1) 5 (100.0) 5 (41.7) 0 (0.0)	^^ X^2^ = 6.19 *p* = 0.07	11	7
					X^2^ = 3.72 *p* = 0.16	X^2^ = 2.55 *p* = 0.28
**Sociodemographic Characteristics**						
**Maternal marital status** Single Married	64 (0.49)	9 (33.3) 22 (56.4)	18 (66.7) 17 (43.6)	X^2^ = 3.41 *p* = 0.08	29	28
					X^2^ = 3.20 *p* = 0.07	X^2^ = 0.29 *p* = 0.59
**Maternal education level** Primary Secondary Tertiary	49 (0.59)	3 (42.9) 17 (47.2) 10 (58.8)	4 (57.1) 19 (52.8) 7 (41.2)	^^ X^2^ = 0.78 *p* = 0.75	29	25
					X^2^ = 0.26 *p* = 0.88	X^2^ = 0.02 *p* = 0.99
**Partner education level** Primary Secondary Tertiary	33 (0.46)	3 (42.9) 22 (66.7) 3 (27.3)	4 (57.1) 11 (33.3) 8 (72.7)	^^ X^2^= 5.65 *p* = 0.06	27	24
					X^2^ = 2.81 *p* = 0.25	X^2^ = 2.17 *p* = 0.34
**Household monthly income (XCD)** Under 1000 1001–2000 2001–3000 Over 3000	41 (0.99)	7 (70.0) 7 (43.8) 7 (41.2) 5 (41.7)	3 (30.0) 9 (56.3) 10 (58.8) 7 (58.3)	X^2^ = 2.56 *p* = 0.49	26	20
					X^2^ = 4.63 *p* = 0.20	X^2^ = 7.24 *p* = 0.07
**Infection Status**						
**Child’s DENV infection status** DENV-positive DENV-negative	60 (0.30)	3 (50.0) 27 (50.0)	3 (50.0) 27 (50.0)	^^ *p* = 1.00	24	22
					X^2^ = 0.67 *p* = 0.41	X^2^ = 0.09 *p* = 0.76
**Child’s ZIKV infection status** ZIKV-positive ZIKV-negative	60 (0.18)	2 (100.0) 28 (48.3)	0 (0.0) 30 (51.7)	^^ X^2^ = 2.01 *p* = 0.49	23	22
					X^2^ = 1.13 *p* = 0.29	X^2^ = 1.03 *p* = 0.31

%s presented represent %s in ZIKV-exposed and unexposed groups. ^^ Fisher’s exact test used to obtain *p*-value when >20% of cells had expected counts of less than 5. Chi-square test was used when <=20% of cells had expected counts of less than 5. ^ Cannot be computed because at least one of the groups is too small (*n* = 0 or *n* = 1) or there are more than two categories t: independent sample *t* test statistic, X^2:^ chi-square test statistic. ^a^ Respiratory distress, meconium aspiration, nuchal cord. ^b^ Asphyxia, meconium aspiration, respiratory distress, mildly/severely depressed APGAR score. ^c^ Resuscitation, jaundice. ^d^ Hydrocephalus, microcephaly, drooping eye, right foot talipes. ^e^ Defined as between 1st and 3rd percentile WHO head circumference for age standards. ^f^ Defined as <1st percentile WHO head circumference for age standards. ^g,h^ Test statistic compares participants who remained in the study vs. those lost to follow-up for the target participant characteristic.

**Table 2:** Cognitive, motor and language neurodevelopmental scores at ages 1, 3 and 4 years between ZIKV-exposed and ZIKV-unexposed children.

Age at Assessment (Neurodevelopment Test)	ZIKV Exposure Group	Cognition			Motor			Language		
		N	Mean (SD)	Test Statistic; *p*	N	Mean (SD)	Test Statistic; *p*	N	Mean (SD)	Test Statistic; *p*
**1 year** **(OX-NDA)**	ZIKV-exposed	30	56.96 (20.76)	*t* = –0.56 *p* = 0.58	30	95.83 (21.87)	*t* = 1.56 *p* = 0.12	29	71.43 (45.24)	*t* = 1.91 *p* = 0.06
	ZIKV-unexposed	34	54.18 (19.16)		34	87.5 (20.83)		32	52.38 (32.14)	
**3 years** **(NEPSY-II)**	ZIKV-exposed	31	8.06 (2.48)	*t* = –0.20 *p* = 0.83	31	7.71 (2.66)	*t* = 0.42 *p* = 0.67	30	7.33 (1.99)	*t* = –1.34 *p* = 0.19
	ZIKV-unexposed	27	8.20 (2.58)		26	7.38 (3.18)		26	8.13 (2.47)	
**4 years** **(NEPSY-II)**	ZIKV-exposed	31	7.95 (2.31)	*t* = –0.77 *p* = 0.44	31	8.52 (2.27)	*t* = -0.15 *p* = 0.88	31	7.95 (1.89)	*t* = 0.08 *p* = 0.93
	ZIKV-unexposed	28	8.42 (2.35)		28	8.61 (2.34)		28	7.90 (2.66)	

*t*: independent sample *t* test statistic.

**Table 3:** Behavioural neurodevelopmental scores at ages 1, 3 and 4 years between ZIKV-exposed and ZIKV-unexposed children.

Age at Assessment (Neurodevelopment Test)	ZIKV Exposure Group	Positive Behaviour			Negative Behaviour			Personal–Social		
		N	Mean (SD)	Test Statistic; *p*	N	Mean (SD)	Test Statistic; *p*	N	Mean (SD)	Test Statistic; *p*
**1 year** **(OX-NDA)**	ZIKV-exposed	30	70.00 (80.00)	*t* = –0.02 *p* = 0.98	30	75.00 (25.00)	*t* = –0.00 *p* = 1.00	–	–	-
	ZIKV-unexposed	30	70.38 (40.00)		30	75.00 (50.00)		–	–	
**3 years** **(NEPSY-II)**	ZIKV-exposed	–	–	–	–	–	–	28	8.86 (3.59)	*t* = –0.30 *p* = 0.76
	ZIKV-unexposed	–	–			–		21	9.14 (2.74)	
**4 years** **(NEPSY-II)**	ZIKV-exposed	–	–	–	–	–	–	30	8.00 (2.78)	*t* = –1.06 *p* = 0.29
	ZIKV-unexposed	–	–			–		26	8.77 (2.61)	

*t*: independent sample *t* test statistic.

**Table 4:** Rates of neurodevelopmental delay at ages 1, 3 and 4 years between ZIKV-exposed and ZIKV-unexposed children.

Age at Assessment (Neuro-Development Test)	ZIKV Exposure Group	Cognition				Motor				Language				Personal–Social			
		Delay	No Delay	Test Statistic; *p*	OR (95% CI)	Delay	No Delay	Test Statistic; *p*	OR (95% CI)	Delay	No Delay	Test Statistic; *p*	OR (95% CI)	Delay	No Delay	Test Statistic; *p*	OR (95% CI)
		N (%), Mean (SD) or Median (IQR)	N (%), Mean (SD) or Median (IQR)			N (%), Mean (SD) or Median (IQR)	N (%), Mean (SD) or Median (IQR)			N (%), Mean (SD) or Median (IQR)	N (%), Mean (SD) or Median (IQR)			N (%), Mean (SD) or Median (IQR)	N (%), Mean (SD) or Median (IQR)		
**1 year** **(OX-NDA)**	ZIKV- exposed	12 (40.0)	18 (60.0)	X^2^ = 3.91 *p* = 0.048	0.36 (0.13 – 1.00)	4 (13.3)	26 (86.7)	^^ *p* = 0.74	0.72 (0.18–2.83)	11 (37.9)	18 (62.1)	X^2^ = 3.67 *p* = 0.06	0.37 (0.13–1.03)	-	-	-	-
	ZIKV- unexposed	22 (64.7)	12 (35.3)			6 (17.6)	28 (82.4)			20 (62.5)	12 (37.5)			-	-	-	-
**3 years** **(NEPSY-II)**	ZIKV- exposed	11 (37.9)	18 (62.1)	X^2^ = 0.00 *p* = 0.97	1.02 (0.33 – 3.11)	13 (44.8)	16 (55.2)	X^2^ = 0.28 *p* = 0.60	0.75 (0.25–2.32)	11 (40.7)	16 (59.3)	X^2^ = 0.44 *p* = 0.51	0.69 (0.23–2.08)	12 (42.9)	16 (57.1)	X^2^ = 1.92 *p* = 0.17	2.40 (0.69–8.39)
	ZIKV- unexposed	9 (37.5)	15 (62.5)			12 (52.2)	11 (47.8)			12 (50.0)	12 (50.0)			5 (23.8)	16 (76.2)		
**4 years** **(NEPSY-II)**	ZIKV- exposed	14 (48.3)	15 (51.7)	X^2^ = 2.65 *p* = 0.10	2.53 (0.82 – 7.86)	9 (31.0)	20 (69.0)	X^2^ = 0.33 *p* = 0.56	0.72 (0.24–2.20)	13 (44.8)	16 (55.2)	X^2^ = 0.23 *p* = 0.63	1.30 (0.44–3.82)	16 (53.3)	14 (46.7)	X^2^ = 2.90 (*p* = 0.09)	2.57 (0.86–7.72)
	ZIKV- unexposed	7 (26.9)	19 (73.1)			10 (38.5)	16 (61.5)			10 (38.5)	16 (61.5)			8 (30.8)	18 (69.2)		

The OX-NDA does not include a measure of the personal–social domain. ^^ Fisher’s exact test used to obtain *p*-value when >20% of cells had expected counts of less than 5. Chi-square test was used when ≤20% of cells had expected counts of less than 5.

**Table 5:** Visual acuity and contrast sensitivity scores, and rates of low vision, at ages 3 and 4 years between ZIKV-exposed and ZIKV-unexposed children.

Age at Assessment	ZIKV Exposure Group	Visual Acuity (VA; LogMAR) ^1^						Contrast Sensitivity (CS; %) ^2^					
		N	VA Score	Group Comparison for VA Score	Low VA	Group Comparison for VA Score	OR (95% CI)	N	CS Score	Group Comparison for CS Score	Low CS	Group Comparison for CS Score	OR (95% CI)
			Mean (SD)	Test Statistic; *p*	N (%)	Test Statistic; *p*			Mean (SD)	Test Statistic; *p*	N (%)	Test Statistic; *p*	
**3 years**	ZIKV-exposed	22	0.26 (0.13)	*t* = 1.19 *p* = 0.24	4 (18.2%)	^^ *p* = 0.69	1.55 (0.31–7.89)	29	59.66 (23.76)	*t* = –0.97 *p* = 0.33	2 (6.9%)	^^ *p* = 0.50	–
	ZIKV-unexposed	24	0.22 (0.11)		3 (12.5%)			25	65.33 (18.58)		0 (0.0%)		
**4 years**	ZIKV-exposed	29	0.17 (0.08)	*t* = -2.02 *p* = 0.051	–	–	–	29	76.88 (20.94)	*t* = 1.13 *p* = 0.26	0 (0.0%)	^^ *p* = 0.22	–
	ZIKV-unexposed	26	0.24 (0.16)		–			26	70.19 (22.87)		2 (7.7%)		

*t*: Independent sample *t* test; ^^ Fisher’s exact test. Group comparisons for low visual acuity at age 4 years were not conducted as norms for the Cardiff vision test are available up to 36 months of age. ^1^ Visual acuity is measured in LogMar; lower scores indicate better visual acuity. ^2^ Contrast sensitivity is measured in percentage (%); lower contrast %s indicate better vision outcomes on this test.

## Data Availability

The data presented in this study are available on reasonable request addressed to the corresponding author.
